# Oxaliplatin/capecitabine or carboplatin/paclitaxel-based preoperative chemoradiation for resectable oesophageal adenocarcinoma (NeoSCOPE): Long-term results of a randomised controlled trial

**DOI:** 10.1016/j.ejca.2021.05.020

**Published:** 2021-08

**Authors:** Somnath Mukherjee, Christopher Hurt, Ganesh Radhakrishna, Sarah Gwynne, Andrew Bateman, Simon Gollins, Maria A. Hawkins, Joanne Canham, Heike I. Grabsch, Stephen Falk, Ricky A. Sharma, Ruby Ray, Rajarshi Roy, Catrin Cox, Nick Maynard, Lisette Nixon, David J. Sebag-Montefiore, Timothy Maughan, Gareth O. Griffiths, Tom D.L. Crosby

**Affiliations:** a Oxford Institute for Radiation Oncology, Oxford University, Oxford, OX3 7DQ, UK; b Department of Oncology, Oxford University Hospital, Oxford University Hospitals NHS Foundation Trust, Oxford, OX3 7LE, UK; c Centre for Trials Research, Cardiff University, Cardiff, CF14 4YS, UK; d Christie Hospital, Christie NHS Foundation Trust, Manchester, M20 4BX, UK; e South West Wales Cancer Centre, Singleton Hospital, Swansea Bay University Health Board, Swansea, SA2 8QA, UK; f Clinical Oncology, University Hospital Southampton, Southampton, SO16 6YD, UK; g North Wales Cancer Treatment Centre, Betsi Cadwaladr University Health Board, Rhyl, LL18 5UJ, UK; h Cancer Institute, University College London, London, WC1E 6DD, UK; i Department of Pathology, Maastricht University Medical Center, Maastricht, the Netherlands; j Leeds Institute of Medical Research, University of Leeds, Leeds, LS9 7TF, UK; k Bristol Haematology and Oncology Centre, University Hospitals Bristol, Bristol, BS2 8ED, UK; l Castle Hill Hospital, Hull University Teaching Hospitals NHS Trust, Hull, HU16 5JQ, UK; m Southampton Clinical Trials Unit, University of Southampton, Southampton, SO16 6YD, UK; n Velindre Cancer Centre, Velindre University NHS Trust, Cardiff, CF14 2TL, UK

**Keywords:** Randomised controlled trial, Neoadjuvant chemoradiotherapy, Surgery, Survival, Oesophageal cancer

## Abstract

**Aim:**

This is the first randomised study to evaluate toxicity and survival outcomes of two neoadjuvant chemoradiotherapy (CRT) regimens for patients with localised oesophageal adenocarcinoma (OAC) or gastro-oesophageal junction (GOJ) adenocarcinoma. The initial results showed comparable toxicity between regimens and pathological complete response (pCR) rate favouring CarPacRT. Herein, we report survival, progression patterns, and long-term toxicity after a median follow-up of 40.7 months.

**Methods:**

NeoSCOPE was an open-label, UK multicentre, randomised, phase II trial. Eighty-five patients with resectable OAC or GOJ adenocarcinoma, ≥cT3 and/or ≥cN1 (TNM v7), suitable for neoadjuvant CRT, were recruited between October 2013 and February 2015.

Patients were randomised to OxCapRT (oxaliplatin 85 mg/m^2^ on Days 1, 15, and 29; capecitabine 625 mg/m^2^ orally twice daily on days of radiotherapy [RT]) or CarPacRT (carboplatin AUC2; paclitaxel 50 mg/m^2^ on Days 1, 8, 15, 22, and 29). RT dose was 45 Gy/25 fractions/5 weeks. Both arms received induction chemotherapy (two cycles oxaliplatin 130 mg/m^2^ on Day 1, capecitabine 625 mg/m^2^ orally twice daily on Days 1–21) before CRT. Surgery was performed 6–8 weeks after CRT.

The primary end-point was pCR. Secondary end-points were toxicity, progression-free survival (PFS), overall survival (OS), and patterns of progression.

**Results:**

Eighty-five patients were recruited from 17 UK centres. The median OS was 41.7 months (95% confidence interval [CI] 19.6 to not reached) in the OxCapRT arm and was not reached in the CarPacRT arm (multivariable hazard ratio [HR] = 0.48, 95% CIs: 0.24–0.95, *P* = 0.035). The median PFS was 32.6 months (95% CIs: 17.1 to not reached) in the OxCapRT arm and was not reached in the CarPacRT arm (multivariable HR = 0.54, 95% CIs: 0.29–1.01, *P* = 0.053). In both arms, the distant progression was twice as common as locoregional progression.

**Conclusions:**

OS and PFS favoured neoadjuvant CarPacRT over OxCapRT. Distant was more common than locoregional progression; therefore, priority should be given to optimising the systemic treatment component.

**Clinical trial information:**

EudraCT Number: 2012-000640-10; ClinicalTrials.gov: NCT01843829.

## Highlights

• Randomised study of neoadjuvant chemoradiotherapy for oesophageal adenocarcinoma.
• The median overall survival was significantly better with CarPacRT.
• Across both arms, the distant was more common than locoregional progression.

## 1 Introduction

Except for early stage disease, treatment by surgery alone confers poor outcome in patients with resectable oesophageal cancer. The CROSS trial showed that neoadjuvant CarPacRT was associated with a doubling of median overall survival (OS) to 49.4 months compared with surgery alone and established a new standard of care [1]. Oxaliplatin has been shown to be comparable in efficacy to cisplatin in advanced gastro-oesophageal cancer and can be conveniently delivered as a 2-h infusion, and oxaliplatin-capecitabine was considered as an international standard of care for advanced gastro-oesophageal adenocarcinoma [2].

NeoSCOPE was a randomised phase II trial that evaluated the efficacy and toxicity of OxCapRT and CarPacRT in the neoadjuvant treatment of patients with locally advanced resectable oesophageal adenocarcinoma (OAC) and assessed the feasibility of safely introducing neoadjuvant chemoradiotherapy (nCRT) into clinical practice in the United Kingdom, where previously neoadjuvant chemotherapy was standard of care. The aim was to ‘pick a winner’ that could be taken forward to a future phase III trial where nCRT would be compared with neoadjuvant chemotherapy.

The primary end-point of the trial, pathological complete response (pCR), together with secondary end-points of acute treatment toxicity, compliance, and complications, was reported in 2017 when all patients had completed surgery [3]. The analysis showed comparable toxicity and postoperative morbidity/mortality in both arms (one death within 30 days of surgery in each trial arm). Although the rate of neutropenia was higher in CarPacRT (9/42 [21.4%] versus 1/38 [2.6%]), this did not lead to higher incidence of neutropenic sepsis/death. The proportion of patients undergoing surgery and proportion of patients with microscopically negative resection margin (viable tumour >1 mm from margin) both favoured CarPacRT (41/43 [95.3%] and 33/41 [80.5%], respectively, versus 36/42 [85.7%] and 26/36 [72.2%] in OxCapRT arm). The pCR rate was also higher in the CarPacRT arm (12/41 [29.3%] versus 4/36 [11.1%]).

Here, for the first time, we report the end-points of OS, progression-free survival (PFS), and patterns of progression, which were analysed when a minimum follow-up of 3 years after surgery for all surviving patients had been achieved.

## 2 Methods

### 2.1 Patients

The design of this multicentre, randomised, open-label, ‘pick a winner’, phase II trial, treatment options, eligibility criteria, and follow-up modalities were previously reported in detail [3,4]. In summary, the trial included patients with the following key eligibility criteria: resectable OAC including Siewert Type 1 or 2 tumour of the gastro-oesophageal junction (GOJ; maximum extension of 3 cm into stomach), with cT stage ≥3 and/or cN stage ≥1 (TNM v7), World Health Organisation performance status 0–1, maximum disease (T + N) length 8 cm, adequate respiratory, cardiac, haematological, renal, and hepatic function, and aged ≥18 years. All patients provided written informed consent.

### 2.2 Randomisation

All patients fulfilling inclusion/exclusion criteria were randomly assigned (1:1) to OxCapRT or CarPacRT by stratified minimisation (by recruiting hospital, cT stage [T1/T2 versus T3/T4], and cN stage [N0 versus N+]) with a random element (80:20) via a centralised computer system.

### 2.3 Procedures

All patients underwent upper gastrointestinal endoscopy with biopsy, staging computed tomography (CT), and positron emission tomography–computed tomography (PET-CT) scan and endoscopic ultrasound (unless contraindicated). Patients in both arms received induction chemotherapy, which consisted of two 3-weekly cycles of oxaliplatin (130 mg/m^2^ intravenously on Day 1) and capecitabine (625 mg/m^2^ orally twice daily from Day 1 to Day 21) before starting chemoradiotherapy (CRT). During the CRT phase, patients randomly assigned to the OxCapRT arm received oxaliplatin (85 mg/m^2^ intravenously on Days 1, 15, and 29) and capecitabine (625 mg/m^2^ orally twice daily on days of radiotherapy [RT]). The CarPacRT regime consisted of carboplatin AUC2 and paclitaxel 50 mg/m^2^ administered intravenously on Days 1, 8, 15, 22, and 29 of RT.

The RT was planned using intravenous contrast CT simulation with minimum of 3-mm CT slices. The RT dose was 45 Gy in 25 daily fractions, delivered Monday to Friday as a 3D, conformally planned single-phase treatment and prescribed according to recommendations of the International Commission on Radiation Units and Measurements (ICRU-50). The trial included rigorous RT quality assurance as previously described [4,5]. Restaging CT/PET-CT was undertaken 4–6 weeks after CRT, and surgery was performed at 6–8 weeks after completion of CRT. Surgical approach was not mandated. Postoperative pathology was reported with Mandard's tumour regression grade (TRG). Treatment toxicities were assessed as per US National Cancer Institute's Common Terminology Criteria for Adverse Events (version 4.03).

Follow-up was undertaken 3 weekly during induction chemotherapy and weekly during the CRT phase. Postoperative follow-up was at 30 days, 6 months, and 12 months after the surgery. Investigations and follow-up beyond 12 months and choice of treatment at relapse were left to the discretion of the treating clinician. Data on events (death and progression) were collected through case report forms.

The trial protocol was approved by the UK Medicines and Healthcare products Regulatory Agency and a Multi-Centre Research Ethics Committee, sponsored by Velindre University NHS Trust and coordinated by the Centre for Trials Research at Cardiff University. The trial was funded by Cancer Research UK (C44694/A14614), who had no role in study design, data collection, data analysis, data interpretation, or publication of the results. The lead authors (S.M., C.H., and T.C.) had full access to the data and final responsibility to submit for publication.

### 2.4 End-points

The primary end-point of the trial was pCR rate. Secondary end-points were feasibility of recruitment, toxicity, perioperative morbidity/mortality, circumferential resection margin positivity rate, and survival. The primary end-point and early toxicity data have been previously reported [3]. Herein we report, for the first time, OS (time-to-event), PFS (time-to-event), and patterns of progression and late toxicity at 6 and 12 months.

### 2.5 Statistical analysis

Data were analysed according to a prespecified statistical analysis plan using the Stata SE 16 statistical package (StataCorp, College Station, Texas 77845 USA). PFS was defined as the interval between randomisation and the earliest occurrence of disease progression resulting in primary (or perioperative) irresectability of disease, locoregional recurrence (after completion of therapy), distant dissemination (during or after completion of treatment), or death from any cause. As in the CROSS trial, this definition for PFS was taken from the modified STEEP criteria for neoadjuvant treatment trials [6]. OS was defined as the interval between randomisation and death from any cause. Patients who were event free were censored at the time they were last known to be event free. We estimated event time distributions with the Kaplan–Meier method and compared OS and PFS with hazard ratios (HRs) from Cox regression in univariable and multivariable models. In the multivariable models, we included all variables thought a priori to have a prognostic effect (including the randomisation stratification variables and others; see Table 1) and included treating centre as a random frailty effect. We tested the proportional hazards assumption of each model with Cox-Snell residuals and Schoenfeld's global test. Three- and 5-year survival were estimated using life tables with actuarial adjustment. Survival analyses were primarily in the intention-to-treat (ITT) population with sensitivity analyses in the per-protocol (PP) population who received at least one cycle of chemotherapy on the allocated CRT regimen. Numbers needed to treat to prevent death were calculated using methods published elsewhere [7]. Toxicity analyses were conducted in the PP population who received surgery and toxicity assessments at the follow-up time points.

**Table 1:** Univariable and multivariable Cox regression overall survival analyses.

Baseline variable		Univariable Cox			Multivariable Cox^a^		
		HR	*P* value	95% CIs	HR	*P* value	95% CIs
Trial arm	OxCapRT	1.00			1.00		
	CarPacRT	0.56	0.079	0.29–1.07	0.48	0.035	0.24–0.95
T stage	1 or 2	1.00			1.00		
	3 or 4	0.63	0.388	0.22–1.80	0.62	0.415	0.20–1.95
N stage	N0	1.00			1.00		
	N+	1.80	0.124	0.85–3.83	1.25	0.586	0.56–2.80
Age (years)	<65	1.00			1.00		
	65+	2.12	0.028	1.09–4.13	2.02	0.052	0.99–4.09
WHO PS	0	1.00			1.00		
	1	1.60	0.241	0.73–3.50	1.37	0.457	0.60–3.14
Tumour length	<6 cm	1.00			1.00		
	≥6 cm	0.98	0.961	0.51–1.88	1.03	0.935	0.53–2.01
Sex	Male	1.00			1.00		
	Female	2.06	0.045	1.02–4.18	2.25	0.037	1.05–4.81

CI, confidence interval; HR, hazard ratios; WHO PS, World Health Organisation performance status.

a Including all variables in this table and treating centre as a shared frailty.

## 3 Results

### 3.1 Study population

**Fig. 1:**
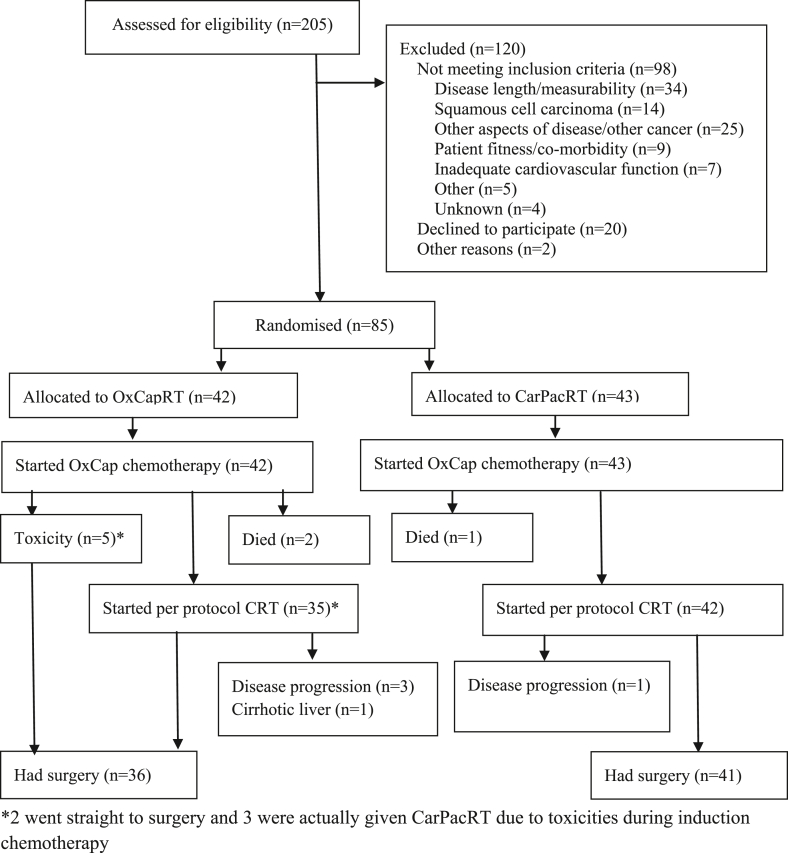
CONSORT flow diagram of trial participants.

Between 10th October 2013 and 12th February 2015, 85 patients were registered into the trial from 17 hospitals across the United Kingdom and 77 underwent surgery (Fig. 1). Patient and tumour baseline characteristics were balanced between the groups (eTable 1 in Supplement 2). The minimum follow-up for surviving patients was 3 years with a median follow-up time of 43.4 (95% confidence interval [CI]: 37.7–53.6) and 51.0 (95% CI: 45.5–54.3) months in the OxCapRT and CarPacRT arm, respectively. At the time of analysis, 37 patients had died (21/42 [50%] in OxCapRT arm and 16/43 [37%] in CarPacRT arm).

### 3.2 Overall survival

**Fig. 2:**
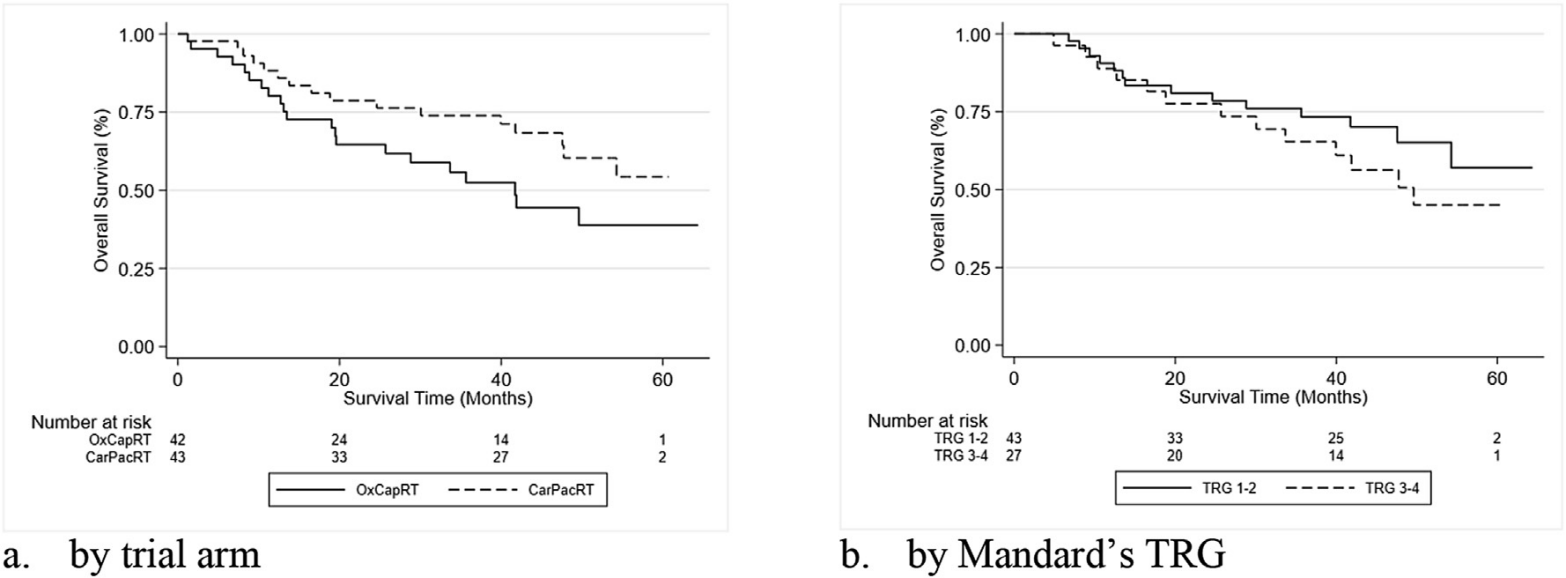
Kaplan–Meier curves for the overall survival.

The analysis of OS in the ITT population (n = 85) is shown in Table 1. At the time of analysis, 48 of 85 patients were alive; 21 of 42 (50%) in the OxCapRT arm, and 27 of 43 (63%) in the CarPacRT arm (Table 2). The median OS was 41.72 months (95% CI 19.58 to not reached) in the OxCapRT arm (Fig. 2a) and was not reached in the CarPacRT arm. Three- and 5-year OS were 52% (95% CI: 35%–67%) and 39% (95% CI: 21%–56%) in the OxCapRT arm and 74% (95% CI: 58%–85%) and 54% (95% CI: 33%–70%) in the CarPacRT arm, respectively. The evidence for this treatment effect favouring CarPacRT was statistically significant in the multivariable analysis (HR = 0.48, 95% CIs: 0.24–0.95, *P* = 0.035). The treatment effect was consistent in magnitude across subgroups (eFig. 1 in Supplement 2). In the PP population (those who started their allocated chemoradiotherapy regimen, n = 77), the treatment effect was of a similar magnitude, although it did not reach statistical significance at the 5% level (multivariable HR = 0.54, 95% CIs: 0.25–1.14, *P* = 0.106). It can also be seen from Table 1 that there was some evidence that lower age and being male were both associated with better survival. Causes of death and 30- and 90-day postsurgery mortality are given in e.Table 2 in Supplement 2 The estimated number of patients who need to be treated with CarPacRT to prevent one additional death at 5 years was 4.1 (95% CI 2.6–53.2).

**Table 2:** Patterns of disease progression.

	OxCapRT		CarPacRT	
All patients	N = 42		N = 43	
	n	%	n	%
Alive and without progression	18	43	24	56
Died before progression detected	8^a^	19	8^b^	19
Progression detected prior to death	16	38	11	26
Locoregional first	4	10	3	7
Metastatic first	10	24	7	16
Both	2	5	1	2
All patient who received allocated CRT regimen and had surgery	N = 31		N = 41	
	n	%	n	%
Alive and without progression	17	55	24	59
Died before progression detected	5^a^	16	7^b^	17
Progression detected prior to death	9	29	10	24
Locoregional first	2	6	3	7
Metastatic first	5	16	6	15
Both	2	6	1	2

a Two oesophageal cancer.

b One oesophageal cancer.

### 3.3 Progression-free survival

**Fig. 3:**
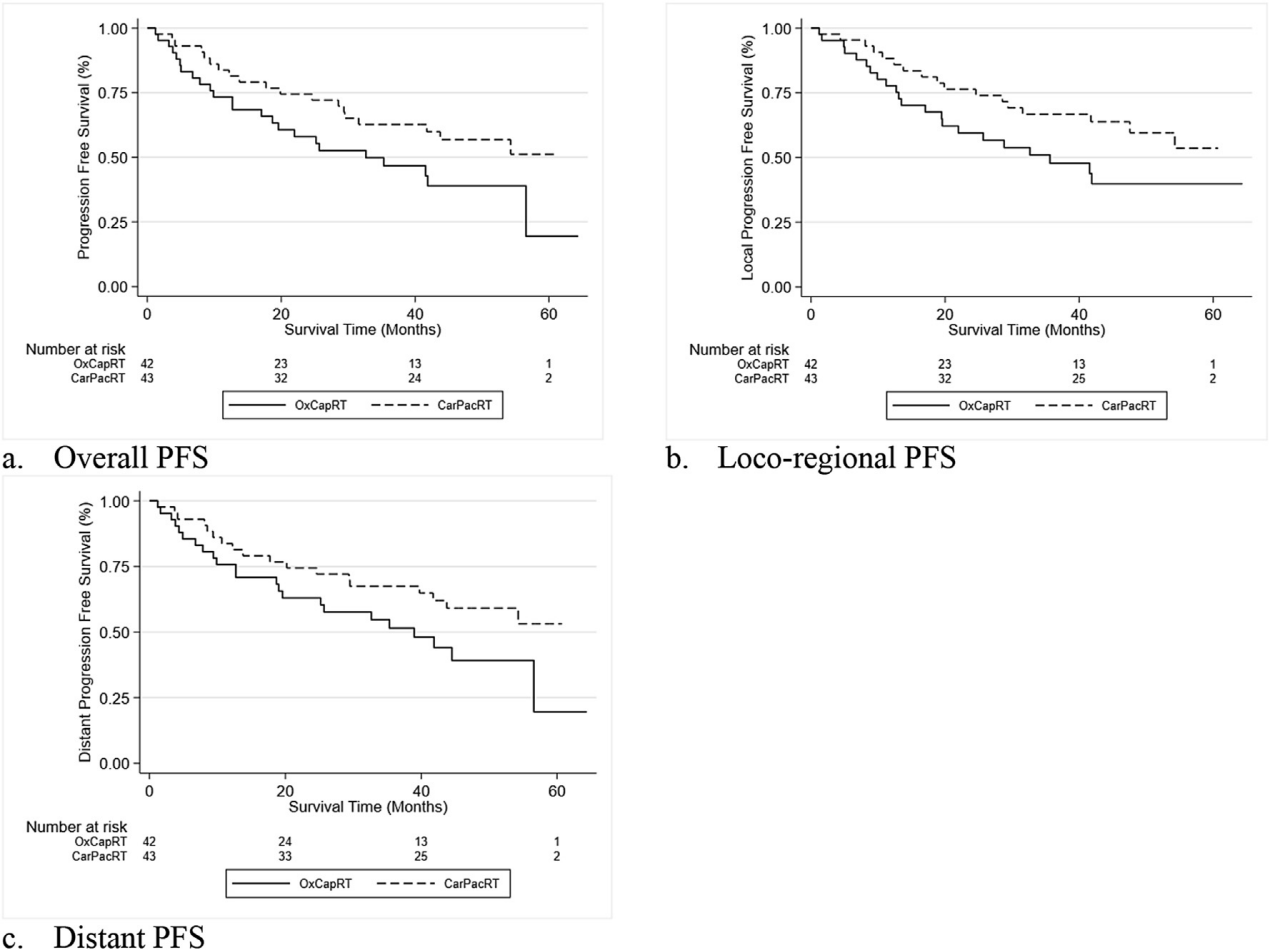
Kaplan–Meier curves for progression-free survival by trial arm.

At the time of analysis, 42 of 85 patients were alive and free of disease (18/42 [43%] in the OxCapRT arm and 24/43 [56%] in the CarPacRT arm). In the ITT population, the median PFS was 32.6 months (95% CIs: 17.1 to not reached) in the OxCapRT arm and was not reached in the CarPacRT arm (Fig. 3a). Proportion of patients progression free at 1 year (68% [95% CI: 52–80] versus 81% [95% CI: 66–90]), 3 years (47% [95% CI: 30–61] versus 63% [95% CI: 47–75]) and 5 years (19% [95% CI: 2–51]) versus 51% [95% CI: 32–67]) favoured CarPacRT. The evidence for this treatment effect favouring CarPacRT was stronger in the multivariable analysis (HR = 0.54, 95% CIs: 0.29–1.01, *P* = 0.053) than the univariable (HR = 0.59, 95% CIs: 0.32–1.07, *P* = 0.084). In the PP population (those who started their allocated chemoradiotherapy regimen, n = 77), the treatment effect was of a similar magnitude (multivariable HR = 0.58, 95% CIs: 0.29–1.19, *P* = 0.137) but did not reach statistical significance.

### 3.4 Patterns of progression

Table 2 summarises patterns of progression, and Fig. 3b and c represents Kaplan–Meier curves for locoregional PFS and distant PFS. Of the 27 patients with disease progression, seven had locoregional site of first progression, 17 relapsed systemically, and three had combined locoregional and systemic progression. In both the ITT population and those patients receiving allocated treatment and surgery, there were approximately twice as many distant than locoregional progressions in both trial arms.

Of the 72 patients who received allocated the CRT regimen and had surgery, 70 were assessable for Mandard's TRG (one as missing and one was ypT0, ypN1). Of these, 43 patients had Mandard's TRG 1 or 2 on resection, and of those, seven patients had a distant recurrence detected first, zero had a local recurrence detected first, eight died before recurrence, and 28 were still alive without recurrence. Twenty-seven patients had Mandard's TRG 3 or 4 on resection (no patients were TRG 5), and of those, four patients had a distant recurrence detected first, seven had a local (or local and distant) recurrence detected first, four died before recurrence, and 12 were still alive without recurrence. The median OS was 49.6 (95% CIs: 30.1 to not reached) in the TRG 3/4 patients and was not reached in the TRG 1/2 patients (HR = 1.49, 95% CIs: 0.70–3.17, *P* = 0.301; Fig. 2b).

### 3.5 Late toxicity

We have previously reported the rates of acute toxicity rate for our patients [3]. eTable 3 in Supplement 2 shows the Grade 3 or 4 toxicities experienced by patients at 6 and 12 months postsurgery. There were more patients who experienced at least one Grade 3 or 4 toxicity at 6 months in the OxCapRT arm: 4/27 (15%) versus 1/38 (3%; λ^2^ = 3.300, *P* = 0.069), but at 12 months, only one patient in each arm had a Grade 3 or 4 toxicity. eTable 4 in Supplement 2 shows that there were fewer persistent Grade 1/2 toxicities observed at both 6 and 12 months postsurgery in the OxCapRT arm: 8/25 (32%) versus 15/34 (44%; λ^2^ = 0.889, *P* = 0.346). Neither comparison was statistically significant.

## 4 Discussion

The mature follow-up of the NeoSCOPE trial demonstrates superior OS in the CarPacRT arm. This only reaches statistical significance in the ITT multivariable analysis, but the magnitude of the treatment effect is similar in the PP analysis and consistent across subgroups and PFS analyses. In addition, there was some evidence that lower age and being male were both associated with better OS. In both groups, systemic progression was more common than locoregional progression. Patients who achieved Mandard TRG 1-2 after CRT had no local recurrences and better survival, but this did not reach statistical significance. A greater proportion of patients on OxCapRT had residual Grade 3/4 toxicities at 6 months postsurgery, but this was not statistically different (and by 12 months, there was no difference between the arms). Broadly, the results are consistent with our initial report, which also favoured the CarPacRT arm by demonstrating a higher pCR and R0 rate with acceptable toxicity.

The CROSS trial established CarPacRT as a standard of care for resectable oesophageal cancer; however, the trial included a mix of squamous cell carcinoma (SCC) and adenocarcinoma (AC), with the magnitude of benefit being much larger in patients with SCC (HR 0.48) [8]. On the other hand, the HR for benefit for AC seen in the CROSS trial (HR 0.73) was similar to trials of pre- and peri-operative chemotherapy (OE02 [9], MAGIC [10], and ACCORD 07 [11]), and it is not clear whether nCRT or neoadjuvant chemotherapy (nCT) is the preferred option. One randomised phase II study of nCT versus nCRT, which predominantly included AC patients (73%), failed to show OS benefit in the CRT group, although R0 resection rates were better and lymph node positivity lower with nCRT [12]. For NeoSCOPE, we therefore only included patients with AC, by far the more common histology in Western countries. We evaluated OxCapRT, as fluoropyrimidine/platinum combination has proven activity in OAC. At the time of designing the trial, oxaliplatin-capecitabine was considered as an international standard of care for advanced gastro-oesophageal adenocarcinoma. We also included induction chemotherapy before CRT, as we postulated that additional cycles of chemotherapy would allow better management of micrometastatic disease.

The pCR and survival outcomes in the CarPacRT arm of NeoSCOPE are similar to those in the AC cohort in the CROSS trial [1,8]. Since the completion of NeoSCOPE, the standard of care for perioperative chemotherapy in gastric/GOJ adenocarcinoma has shifted to the triple combination of 5FU, oxaliplatin, and docetaxel (FLOT), which demonstrated OS superiority over epirubicin, cisplatin, and capecitabine (ECX) [13]. Patients in the CarPacRT arm of NeoSCOPE were exposed to the triple combination of fluoropyrimidine, platinum, and taxane (although sequentially rather than concurrently), and this may be one of the reasons that accounted for superior survival seen in that treatment arm. A randomised control trial of FLOT versus CarPacRT in the neoadjuvant treatment of oesophageal cancer is currently underway (ESOPEC trial) [14].

The pattern of progression (overall 32% [27/85], locoregional progression 11.8% [10/85], and systemic progression 23.5% [20/85]) is similar to patterns of progression seen in CROSS trial [15]. Despite the use of induction chemotherapy, there was a predominance of systemic relapse in this phase 2 study. Although the study was not designed to demonstrate the added benefit of induction systemic therapy, the predominance of systemic failures raises the potential need for ‘better’ rather than ‘more’ chemotherapy. Sequential integration of induction FLOT followed by CarPacRT can potentially lead to more effective control of both systemic and local components of oesophageal cancer and could be tested in future trials; however, unselective use of systemic agents is unlikely to lead to step-change improvement in outcomes. However, careful consideration of the potential incremental risk to patient treatment–related morbidity with increasing systemic agents needs to be taken into account. In our study, we have previously reported our acute toxicity rates [3]. It is noteworthy that the rate of febrile neutropenia in our cohort was 0 in the induction chemotherapy arm and 2.4% in the CarPacRT, which is significantly lower than noted in patients who received FLOT (51%) chemotherapy [14]. It may be that sequential FLOT followed by CarPacRT reduces the rate of cumulative myelosuppressive toxicity by potentially reducing the number of cycles of FLOT chemotherapy required to maintain patient responses and clinical outcomes. The risk of myelosuppression with this sequential approach may be further mitigated with the use of prophylactic haemopoietic growth factors and newer radiation technologies such as proton beam therapy [16].

With our evolving knowledge of the genetic landscape of OAC, we may be able to identify actionable targets, allowing personalisation of treatment strategies based on individual tumour profiles [17].

### 4.1 Limitations and strengths

This is the first randomised study to have compared two preoperative chemoradiation regimens in a purely adenocarcinoma cohort. This trial was not powered to compare survival between arms, and OS was a secondary end-point. Of the 42 patients randomised to the OxCapRT arm, only 36 underwent surgery (compared with 41 of 43 randomised patients in the CarPacRT arm) as more patients had disease progression (3 versus 1) and toxic death (2 versus 1) in the OxCapRT arm. Moreover, three patients crossed over to CarPacRT arm because of toxicity during induction chemotherapy. However, the OS results were consistent in ITT and PP analyses. In addition, we did not collect data regarding what proportion of locoregional progression occurred within the surgical and RT fields, although we did note that the majority of the progressions were distant rather than locoregional.

## 5 Conclusions

NeoSCOPE demonstrated that in patients with resectable OAC or GOJ adenocarcinoma, OS and PFS favoured neoadjuvant CarPacRT over OxCapRT. Across both arms, distant was more common than locoregional progression, suggesting priority should be given to optimising the systemic component of treatment.

## Authors’ contributions

T.C., C.H., S.M. contributed to conceptualisation. C.H. contributed to methodology. C.C. and C.H. contributed to formal analysis. S.M., G.R., S.G., A.B., S.G., M.A.H., H.I.G., S.F., R.A.S., R.R., and N.M. contributed to investigation. C.C. contributed to data curation. S.M., C.H., and T.C. wrote the original article. All authors reviewed and edited the article. C.C. and C.H. contributed to visualisation. T.C., C.H., S.M. contributed to supervision. J.C., L.N., and R.R. contributed to project administration. T.C., C.H., S.M., H.G., A.B., S.G., G.R., R.S., and D.S.-M. contributed to funding acquisition.

## Funding

This work was supported by 10.13039/501100000289Cancer Research UK (grant number: C44694/A14614) but they played no role in the study. MH is supported by funding from the NIHR Biomedical Research Centre at 10.13039/501100008721University College London Hospitals NHS Foundation Trust and 10.13039/501100000765University College London.

## Conflicts of interest statement

The authors declare the following financial interests/personal relationships which may be considered as potential competing interests: RAS is an employee of and holds stock in Varian Medical Systems. All other authors have declared no conflicts of interest.
