# Supplementary material for: Oxaliplatin/capecitabine or carboplatin/paclitaxel-based preoperative chemoradiation for resectable oesophageal adenocarcinoma (NeoSCOPE): Long-term results of a randomised controlled trial
Source: Eur J Cancer. 2021 Aug;153:153–61. doi: 10.1016/j.ejca.2021.05.020 (PMC8330696; doi:10.1016/j.ejca.2021.05.020)

**Supplemental Online Content**

**Mukherjee S, Hurt C, Radhakrishna G, et al. Oxaliplatin/capecitabine or carboplatin/paclitaxel based pre-operative chemoradiation for resectable oesophageal adenocarcinoma (NeoSCOPE): long-term results of a randomised controlled trial.**

eTable 1. Baseline characteristics

eTable 2: Number and causes of death

eTable 3: Grade 3/4 toxicities observed at 6 or 12 months post surgery

eTable 4: Persistent grade 1/2 toxicities observed at both 6 and 12 months post surgery in individual patients

eFigure 1: Hazard ratio forest plots for treatment effect on overall survival, by baseline characteristics

**eTable 1. Baseline characteristics**

|  |  | **OxCapRT (*N=42*)** | | **CarPacRT (*N=43*)** | |
| --- | --- | --- | --- | --- | --- |
|  |  | ***n*** | **%** | ***n*** | **%** |
| Sex | Male | *36* | 85.7 | *33* | 76.7 |
|  | Female | *6* | 14.3 | *10* | 23.3 |
| Age | Median (IQR, Range) | 65 (58-72, 46-77) | | 64 (61-68, 29-76) | |
| cT stage | T2 | *6* | 14.3 | *3* | 7.0 |
|  | T3 | *36* | 85.7 | *37* | 86.0 |
|  | T4a | *0* | 0.0 | *3* | 7.0 |
| cN stage | N0 | *12* | 28.6 | *16* | 37.2 |
|  | N1 | *21* | 50.0 | *20* | 46.5 |
|  | N2 | *8* | 19.0 | *6* | 14.0 |
|  | N3 | *1* | 2.4 | *1* | 2.3 |
| Site of predominant tumour | Middle third (24-<32cm) | *6* | 14.3 | *2* | 4.7 |
|  | Lower third (32-40cm) | *32* | 76.2 | *39* | 90.7 |
|  | Missing | *4* | 9.5 | *2* | 4.7 |
| Time from staging scan to randomization (days) | Median (IQR, Range) | 27 (19-39, 8-56) | | 28 (23-34, 2-51) | |
| Maximum total disease length from EUS, PET and CT | Median (IQR, Range) | 5.85 (4.7-6,2-8) | | 5.7 (5-7, 2-8.3) | |
| WHO Performance Status | 0 | *37* | 88.1 | *35* | 81.4 |
|  | 1 | *5* | 11.9 | *8* | 18.6 |
| Time from randomization to start of treatment (days) | Median (IQR, Range) | 4 (2-6, 0-18) | | 4 (3-6, 0-14) | |

**eTable 2: Number and causes of death**

|  | **OxCapRT (n=42)** | | **CarPacRT (n=43)** | |
| --- | --- | --- | --- | --- |
|  | **n** | **%** | **n** | **%** |
| **Number of deaths** | 21 | 50 | 16 | 37 |
| Oesophageal cancer | 13 | 62 | 8 | 50 |
| Chemotherapy related | 1 | 5 | 0 | 0 |
| Radiotherapy related | 0 | 0 | 1 | 6 |
| Surgery related | 0 | 0 | 0 | 0 |
| Bowel ischaemia | 0 | 0 | 1 | 6 |
| Heart failure | 1 | 5 | 0 | 0 |
| Respiratory failure | 1 | 5 | 0 | 0 |
| Heart disease | 1 | 5 | 0 | 0 |
| Multiple organ failure | 2 | 1 | 0 | 0 |
| Bronchopneumonia | 0 | 0 | 2 | 13 |
| Diarrhoea and acute ischaemic leg | 1 | 5 | 0 | 0 |
| Atypical meningioma grade II brain | 0 | 0 | 1 | 6 |
| Acute pancreatitis | 0 | 0 | 1 | 6 |
| Hospital acquired pneumonia | 0 | 0 | 1 | 6 |
| Other: Unknown | 1 | 5 | 1 | 6 |
| **30-d post-operative mortality** | 1 | 2 | 1 | 2 |
| **90-d post-operative mortality** | 2 | 5 | 1 | 2 |

**eTable 3: Grade 3/4 toxicities observed at 6 or 12 months post surgery**

|  | **6 months timepoint** | | | | **12 months timepoint** | | | |
| --- | --- | --- | --- | --- | --- | --- | --- | --- |
|  | **OxCapRT** | | **CarPacRT** | | **OxCapRT** | | **CarPacRT** | |
|  | **n** | | **n** | | **n** | | **n** | |
| Started per protocol CRT | 35 | | 42 | | 35 | | 42 | |
| Did not have surgery | 4 | | 1 | | 4 | | 1 | |
| Died/lost to follow up before toxicity timepoint | 4 | | 3 | | 6 | | 7 | |
| Toxicity assessments conducted at timepoint | N=27 | | N=38 | | N=25 | | N=34 | |
|  | **n** | **%** | **n** | **%** | **n** | **%** | **n** | **%** |
| Patients with any of the below | 4 | 15 | 1 | 3 | 1 | 4 | 1 | 3 |
| **Cardiac disorders** |  |  |  |  |  |  |  |  |
| Constrictive pericarditis | 1 | 4 | 0 | 0 | 0 | 0 | 0 | 0 |
| **Gastrointestinal** |  |  |  |  |  |  |  |  |
| Abdominal pain | 0 | 0 | 0 | 0 | 1 | 4 | 0 | 0 |
| Dyspepsia | 1 | 4 | 0 | 0 | 0 | 0 | 0 | 0 |
| Gastroesophageal reflux | 0 | 0 | 0 | 0 | 0 | 0 | 0 | 0 |
| Nausea/Vomiting | 1 | 4 | 0 | 0 | 0 | 0 | 0 | 0 |
| **General disorders** |  |  |  |  |  |  |  |  |
| Fatigue | 1 | 4 | 0 | 0 | 0 | 0 | 0 | 0 |
| **Investigations** |  |  |  |  |  |  |  |  |
| Increased alkaline phosphate | 0 | 0 | 0 | 0 | 0 | 0 | 1 | 3 |
| **Metabolism** |  |  |  |  |  |  |  |  |
| Anorexia | 1 | 4 | 0 | 0 | 0 | 0 | 0 | 0 |
| **Nervous system** |  |  |  |  |  |  |  |  |
| Peripheral neuropathy | 1 | 4 | 0 | 0 | 0 | 0 | 0 | 0 |
| **Vascular** |  |  |  |  |  |  |  |  |
| Thromboembolic events | 0 | 0 | 1 | 3 | 0 | 0 | 0 | 0 |

**eTable 4: Persistent grade 1/2 toxicities observed at both 6 and 12 months post surgery in individual patients**

|  | **OxCapRT** | | **CarPacRT** | |
| --- | --- | --- | --- | --- |
|  | **n** | | **n** | |
| Started per protocol CRT | 35 | | 42 | |
| Did not have surgery | 4 | | 1 | |
| Died/lost to follow up before 12 months | 6 | | 7 | |
| Toxicity assessments conducted at 6 and 12 months | N=25 | | N=34 | |
|  | **n** | **%** | **n** | **%** |
| Patients with any of the below | 8 | 32 | 15 | 44 |
| **Blood and lymphatic system disorders** |  |  |  |  |
| Anaemia | 1 | 4 | 2 | 6 |
| **Gastrointestinal** |  |  |  |  |
| *Patients experiencing any in this category* | 3 | 12 | 7 | 21 |
| Abdominal pain | 0 | 0 | 3 | 9 |
| Constipation | 1 | 4 | 0 | 0 |
| Diarrhoea | 0 | 0 | 1 | 3 |
| Dumping syndrome | 1 | 4 | 0 | 0 |
| Gastroesophageal reflux/dyspepsia | 1 | 4 | 2 | 6 |
| Nausea/Vomiting | 0 | 0 | 3 | 9 |
| **General disorders** |  |  |  |  |
| Fatigue | 3 | 12 | 6 | 18 |
| **Investigations** |  |  |  |  |
| Blood bilirubin increased | 0 | 0 | 1 | 3 |
| Platelet decrease | 0 | 3 | 1 | 3 |
| **Metabolism** |  |  |  |  |
| Hypoalbuminemia | 0 | 0 | 2 | 6 |
| **Musculoskeletal and connective tissue disorders** |  |  |  |  |
| Arthritis | 0 | 0 | 1 | 3 |
| **Nervous system** |  |  |  |  |
| Peripheral neuropathy | 3 | 12 | 1 | 3 |
| **Respiratory** |  |  |  |  |
| Bronchiectasis | 0 | 0 | 1 | 3 |
| Cough | 1 | 4 | 1 | 3 |
| Dyspnoea | 1 | 4 | 2 | 6 |
| **Surgical and medical procedures** |  |  |  |  |
| Weak voice (damaged at surgery) | 0 | 0 | 1 | 3 |

**eFigure 1: Hazard ratio forest plots for treatment effect on overall survival, by baseline characteristics**


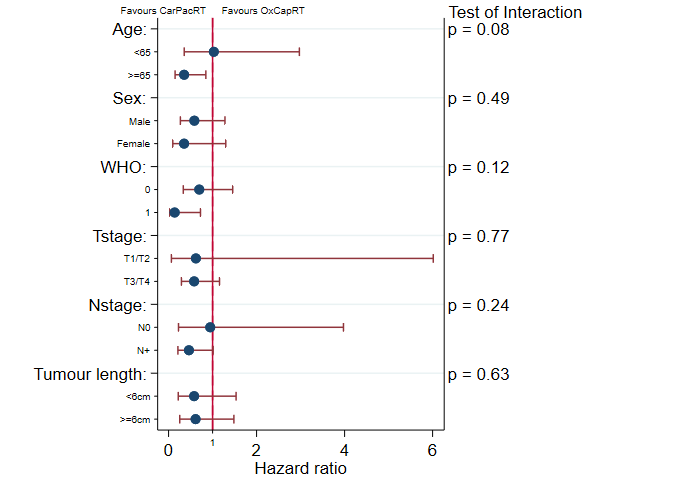

Supplement: Multimedia component 1 [file mmc1.docx]
